# Targets and mechanisms of *Alpinia oxyphylla* Miquel fruits in treating neurodegenerative dementia

**DOI:** 10.3389/fnagi.2022.1013891

**Published:** 2022-11-30

**Authors:** Peng Zeng, Yuan-Cheng Liu, Xiao-Ming Wang, Chao-Yuan Ye, Yi-Wen Sun, Hong-Fei Su, Shuo-Wen Qiu, Ya-Nan Li, Yao Wang, Yan-Chun Wang, Jun Ma, Man Li, Qing Tian

**Affiliations:** ^1^Key Laboratory of Neurological Disease of National Education Ministry, School of Basic Medicine of Tongji Medical College, Huazhong University of Science and Technology, Wuhan, China; ^2^Department of Histology and Embryology, School of Basic Medicine, Hengyang Medical College, University of South China, Hengyang, China; ^3^College of Acupuncture and Orthopedics, Hubei University of Chinese Medicine, Wuhan, China

**Keywords:** neurodegenerative dementia, *Alpinia oxyphylla* Miquel, acetylcholinesterase, amyloid-β, tau

## Abstract

The dried and ripe fruits of *Alpinia oxyphylla* and ripe fruits of *Alpinia oxyphylla* Miquel (AO) have the effects of tonifying kidney-essence and nourishing intelligence and thus have been widely used in treating dementia. Alzheimer’s disease (AD) is a typical form of neurodegenerative dementia with kidney-essence deficiency in Traditional Chinese Medicine (TCM). So far, there is a lack of systematic studies on the biological basis of tonifying kidney-essence and nourishing intelligence and the corresponding phytochemicals. In this study, we investigated the targets of AO in tonifying kidney-essence and nourishing intelligence based on the key pathophysiological processes of neurodegenerative dementia. According to ultra-high-performance liquid chromatography with triple quadrupole mass spectrometry data and Lipinski’s rule of five, 49 bioactive phytochemicals from AO were identified, and 26 of them were found to target 168 key molecules in the treatment of neurodegenerative dementia. Nine phytochemicals of AO were shown to target acetylcholinesterase (ACHE), and 19 phytochemicals were shown to target butyrylcholinesterase (BCHE). A database of neurodegenerative dementia with kidney-essence deficiency involving 731 genes was constructed. Furthermore, yakuchinone B, 5-hydroxy-1,7-bis (4-hydroxy-3-methoxyphenyl) heptan-3-one (5-HYD), oxyhylladiketone, oxyphyllacinol, butyl-β-D-fructopyranoside, dibutyl phthalate, chrysin, yakuchinone A, rhamnetin, and rhamnocitrin were identified as the key phytochemicals from AO that regulate the pathogenesis of neurodegenerative dementia in a multitargeted manner. The approach of studying the pharmacological mechanism underlying the effects of medicinal plants and the biological basis of TCM syndrome may be helpful in studying the translation of TCM.

## Introduction

*Alpinia oxyphylla* Miquel (AO, simplified as 益智仁 in Chinese and meaning “the intelligence-enhancing nut”) is an important medicinal plant seed with a long history of clinical use in Traditional Chinese Medicine (TCM). As one of the four famous medical plants of South China, AO is mainly used to warm the kidney-*qi* and secure the kidney-essence. In terms of clinical use, AO has been widely used in the treatment of neurodegenerative dementia (Wang M. et al., 2018; Zhang et al., 2018; Shi et al., 2020; Bian et al., 2021; Li J. et al., 2021). The common types of neurodegenerative dementia include Alzheimer’s disease (AD), frontotemporal lobar degeneration (FTLD), dementia with Lewy bodies (DLB), and Parkinson’s disease (PD). The theory of TCM believes that kidney-essence deficiency is the internal mechanism underlying neurodegenerative dementia and that AO treats neurodegenerative dementia mainly by tonifying kidney-essence. However, at present, there is a lack of systematic research on the biological basis of kidney-essence deficiency, the targets through which kidney-tonifying herbs exert their effects, and the corresponding phytochemical components that target these molecules.

Among the 75 herbal decoctions and a total of 122 herbal medicines used for tonifying kidney-essence, AO is the most frequently used herb; it has been used 56 times (Yao and Yuejia, 2022). AO is the primary member of several TCM decoctions used for treating dementia, including *Yizhi Xingnao Decoction* (益智醒脑汤) and *Yizhi Decoction* (益智汤). A clinical study showed that *Xingnao Yizhi Granules* (醒脑益智颗粒) were more effective than piracetam (primarily used to improve memory) in the treatment of AD, and there was no difference in adverse reactions between *Xingnao Yizhi Granules* and piracetam (Li J. et al., 2021). Compared with donepezil (a cholinesterase inhibitor) alone, the combination of donepezil and *Yizhi Xingnao Decoction* is more effective in treating AD (Zhang, 2015). Recent neuropharmacological studies have revealed the anti-oxidant, anti-inflammatory, anti-apoptotic, and nerve cell proliferation-promoting effects of AO (Qi et al., 2019; Li J. et al., 2021). Notably, there is currently no report on the toxicities and side effects of AO and its compounds. Acute toxicity studies in rats showed that a single dose of the ethanol extract of AO and its dichloromethane fraction (1,000 mg/kg body weight) administered by gavage did not cause any symptoms of toxicity or mortality (Zhang et al., 2017). Therefore, the targets and mechanisms of AO, which is an important traditional herbal medicine for treating dementia, are worthy of systematic study.

Alzheimer’s disease is the most common form of dementia, accounting for 60-80% of dementia cases; AD is characterized by senile plaques (SPs) that form due to the aggregation of extracellular amyloid-β (Aβ) peptides, neurofibrillary tangles (NFTs) that are composed of intracellular hyperphosphorylated tau (hp-tau), neuroinflammation and loss of memory-related neurons (Hinz and Geschwind, 2017; Islam and Tabrez, 2017; Iyaswamy et al., 2022; Wang et al., 2022; Yang C. B. et al., 2022; Zhu et al., 2022). Pharmacological studies have shown that AO can inhibit the production of Aβ and hyperphosphorylation of tau, thereby affecting antioxidant and anti-inflammatory processes, promoting neuronal proliferation, and inhibiting apoptosis. In an Aβ_25–35_-induced mouse model of AD, administration of *Yizhi Xingnao Decoction* (18 g crude drug/kg/day) by gavage for 4 weeks inhibited tau hyperphosphorylation through the phosphatidylinositol 3-kinase (PI3K)/protein kinase B (Akt) pathway and promoted Aβ degradation by increasing the levels of insulin-degrading enzyme (IDE) and neprilysin (NEP), thereby improving the spatial memory of the mice (Yin et al., 2020; Yang C. et al., 2022). When the *n*-butanol extract of AO (180 mg/kg and 360 mg/kg) was administered by gavage for 20 days, the expression of β-secretase (BACE1) was downregulated in mice with Aβ_1–42_-induced AD, which in turn reduced the level of Aβ in the hippocampus (Shi et al., 2015; Bian et al., 2021). The ethanol extract of AO exhibited a strong ability to inhibit tau hyperphosphorylation at the threonine 202, threonine 231, and serine 396 sites in a glycogen synthase kinase 3 (GSK3β)-dependent manner (Li J. et al., 2021). Furthermore, continuous administration of different doses of AO volatile oil by gavage for 7 days inhibited the apoptosis of the neurons in the substantia nigra of mice with PD (Huang et al., 2011). In an animal model of lipopolysaccharide-induced dementia, intragastric administration of AO extracts (360 mg/kg) for 2 weeks improved cognitive impairment and altered the levels of Aβ_1–42_ and hp-tau (Wang Y. et al., 2018). Although these studies showed that AO might target several pathological changes related to neurodegenerative dementia, a comprehensive understanding of the phytochemicals of AO that target the specific pathophysiological processes of neurodegenerative dementia is still lacking.

As an important traditional Chinese medicine for the treatment of dementia, AO can affect the pathophysiological process of multiple neurodegenerative dementias through multi-component and multi-target. Therefore, we choose AO as a drug for the treatment of AD and analyze its corresponding phytochemical components. Terpenoids, diarylheptanoids, and flavonoids are three major types of phytochemicals that have been identified in AO. Most terpenoids are sesquiterpenoids, such as nootkatone, oxyphyllone E, and oxyphyllol B (Zhang et al., 2018). This study aimed to understand the biological basis of kidney-essence deficiency in neurodegenerative dementia and the targets by which AO exerts its effects based on several important pathophysiological processes related to neurodegenerative dementia, including acetylcholine (ACh) metabolism, Aβ aggregation, tau accumulation, neuroinflammation, and neuron loss. In terms of the AO material basis, the bioactive phytochemicals of AO were identified based on recent highly reliable data obtained by ultra-high-performance liquid chromatography with triple quadrupole mass spectrometry (UHPLC-MS/MS) (Xu et al., 2012; Shi et al., 2014; Zhang et al., 2015; Sun et al., 2016; Chen Y. et al., 2020; Ying et al., 2021). Physicochemical and ADMET studies showed that the compounds that were most compliant with Lipinski’s rule of five (RO5) could penetrate the blood–brain barrier (BBB) to high degrees and exhibited good oral bioavailability (Mohd et al., 2020). Therefore, we considered RO5-compliant phytochemicals from AO to be bioactive phytochemicals, and 49 bioactive phytochemicals in AO were identified. Toxicological safety evaluation bridges the gap between preclinical and clinical studies, and it has important implications for human drug safety. Therefore, we also evaluated the toxicological parameters of the phytochemicals in AO. Twenty-six bioactive phytochemicals in AO were found to target 168 key molecules associated with neurodegenerative dementia. Among them, chrysin, pinocembrin, pinostrobin, rhamnocitrin, and tectochrysin target both acetylcholinesterase (ACHE) and butyrylcholinesterase (BCHE). Yakuchinone B, 5-hydroxy-1,7-bis (4-hydroxy-3-methoxyphenyl) heptan-3-one (5-HYD), oxyhylladiketone, oxyphyllacinol, butyl-β-D-fructopyranoside, dibutyl phthalate, chrysin, yakuchinone A, rhamnetin, and rhamnocitrin were identified as the key phytochemicals of AO that regulate the pathogenesis of neurodegenerative dementia in a multitargeted manner. The approach of studying the pharmacological mechanisms underlying the effects of medicinal plants and the biological basis of TCM syndrome may be helpful in studying the translation of TCM.

## Materials and methods

### Determination of the phytochemicals in *Alpinia oxyphylla* Miquel and evaluation of their pharmacological parameters

We retrieved the phytochemicals in the dried fruits of AO by searching PubMed^1^, and a total of 64 phytochemicals in AO fruits were identified by recent studies that used UHPLC-MS/MS (Xu et al., 2012; Shi et al., 2014; Zhang et al., 2015; Sun et al., 2016; Chen Y. et al., 2020; Ying et al., 2021). Each compound’s SMILES was downloaded from the PubChem database^2^ (Kim et al., 2021) or outlined and exported by ChemDraw Ultra 8.0 software. We used the HobPre web server^3^ (Wei et al., 2022) to predict human oral bioavailability (HOB), which is a key factor in determining the fate of new drugs in clinical trials. Briefly, canonical SMILES were input into the HobPre web server, and the cut-off was set to 50%. RO5, which includes a molecular weight (MW) < 500, number of hydrogen bond donors (Hdon) ≤ 5, number of hydrogen bond acceptors (Hacc) ≤ 10, lipid-water partition coefficient (LogP) ≤ 5 and number of rotatable bonds (Rbon) ≤ 10, is an accepted standard for orally applicable drugs. Here, we used the SwissADME web tool^4^ (Daina et al., 2017) to assess the absorption or permeation of compounds based on RO5. Phytochemicals in AO that were consistent with RO5 were considered bioactive phytochemicals for follow-up studies.

### Prediction of the toxicity of phytochemicals

Toxicological evaluations of phytochemicals are critical for drug development. ProTox-II^5^ (Banerjee et al., 2018) was used to evaluate the toxicities of the identified phytochemicals from AO. ProTox-II extracts various toxicological parameters from the compounds’ SMILES representation to predict acute oral toxicity (LD50, mg/kg) and several toxicity end points, including carcinogenicity, immunotoxicity, mutagenicity, and cytotoxicity, and it ranks the acute toxicity of each compound in six classes, namely, Fatal (Class-1), Fatal (Class-2), Toxic (Class-3), Harmful (Class-4), May be harmful (Class-5), and Non-toxic (Class-6). According to previous research (Wei et al., 2021), a compound in an acute toxicity class lower than Class-3 and with more than three active toxicity end points is considered to be toxic.

### Identification of the potential targets of *Alpinia oxyphylla* Miquel

Based on the molecular similarity, the SwissTargetPrediction web server^6^ (Daina et al., 2019) was used to identify the potential targets of the bioactive phytochemicals of AO. Briefly, the SMILES were input into SwissTargetPrediction, and the target species was set to *Homo sapiens*. Subsequently, target information was collected and organized in Microsoft Excel software (version 2019, Microsoft, Redmond, WA, United States).

### Identification of differentially expressed genes in the hippocampus of patients with Alzheimer’s disease

Gene expression data from the AD dataset (GSE5281) (Liang et al., 2007) were collected from the Gene Expression Omnibus (GEO) database^7^; these data were obtained with the GPL570 platform (Affymetrix Human Genome U133 Plus 2.0 Array). This dataset included 161 human brain samples, and this study focused on 23 samples from the hippocampus (13 control samples and 10 AD samples). The hippocampus is the main brain region affected in the early stage of AD. The limma package in R version 3.6.3 was utilized to identify the DEGs in the hippocampus of patients with AD with cut-offs of adjusted *p*-value < 0.05 and fold change > 2. A volcano map was produced using the R package ggplot2 to show the criteria for selecting DEGs. Heatmaps were created with the R package ComplexHeatmap (version 2.2.0). We also assessed the clustering of hippocampal samples from patients with AD using principal component analysis (PCA). All the software analyses used in this study were performed on Windows (version 10).

### Screening potential targets of *Alpinia oxyphylla* Miquel in the treatment of Alzheimer’s disease

We use the following three strategies to identify the targets of AO in the treatment of AD. First, the “CFG rank” module of the AlzData database^8^ (Xu et al., 2018) was used to screen the targets of AO that are involved in Aβ and tau pathology (key neuropathological hallmarks of AD pathology). Second, 384 genes involved in Alzheimer disease pathway (hsa05010) were downloaded directly from the KEGG database^9^, and then, the intersection of Alzheimer’s disease pathway genes and the potential targets of AO was identified. Third, we performed Disease Ontology (DO) enrichment analysis to determine human diseases in which targets of AO are involved. Based on DO enrichment analysis, we screened targets of AO that are relevant for AD treatment. In this research, DO enrichment analysis was performed by the DOSE Bioconductor package in R (Yu et al., 2015). The Benjamini–Hochberg (BH) method was used to adjust the *p*-value, and only enriched DO terms with an adjusted *p*-value < 0.05 were considered. Rich factor refers to the ratio of gene numbers to all gene numbers annotated in this term. The top 20 DO terms were selected and visualized as a bubble chart using an online tool^10^ (Zeng et al., 2021). The overlap of targets of AO in the treatment of AD was visualized using a UpSetR plot. AlzData’s “Single Cell Expression” module contains gene expression data of different cell types from single-cell RNA-seq analysis of human brain samples (GSE67835), so we used this module to analyze the cell types in which the molecules that are targeted by AO in the treatment of AD are expressed.

### Protein-protein interaction network construction and analysis

Protein-protein interaction networks were constructed using the STRING database (version 11.5^11^) and visualized by Cytoscape software (version 3.7.1). In this study, medium confidence (minimum required interaction score > 0.4) was the selection criterion to construct the PPI network. The Network Analysis plugin of Cytoscape was used to analyze the degree values of the nodes in the network of targets of AO in the treatment of AD, and the Top 30 targets were selected and identified as core targets and were ordered by degree. The higher a protein’s degree is, the more important the protein is in the PPI network.

### Screening of phytochemicals in *Alpinia oxyphylla* Miquel with anticholinesterase activity

ACh, which is an important neurotransmitter for learning and memory, has been implicated in aging-related dementia, in which substantial impairment of hippocampus-dependent cognition is observed. In the brain, ACh predominantly undergoes hydrolysis by ACHE (approximately 80%) and also by BCHE. Decreased cholinergic signaling is a characteristic of AD and vascular dementia, and countering this decreased signaling is one major therapeutic strategy used in the treatment of these diseases (Tomassoni et al., 2012). In AD, decreased ACHE enzymatic activity and increased BCHE activity were also observed in brain regions with increased Aβ and hp-tau deposits (Perry et al., 1978). BCHE compensates for the loss of ACHE and serves as the main enzyme that mediates ACh deactivation in the AD brain. Consequently, the administration of agents that target both ACHE and BCHE is thought to enhance therapeutic efficiency. We used Venny 2.1^12^ to screen the phytochemicals in AO that target ACHE and BCHE as potential compounds for the symptomatic treatment of dementia.

### Screening of phytochemicals of *Alpinia oxyphylla* Miquel that target key pathological features of neurodegenerative dementia

Based on Alzheimer’s disease pathway (hsa05010), we analyzed the phytochemicals of AO and their targets that are involved in the production and degradation of Aβ, such as APP, α-secretase, β-secretase, γ-secretase, MME, and IDE. Based on Alzheimer’s disease pathway (hsa05010), Gene Ontology (GO) enrichment analysis and a literature search, we screened AO targets and the corresponding phytochemicals that are involved in the regulation of tau phosphorylation, tau degradation, neuroinflammation and neuron death. GO enrichment analysis was conducted with the ClusterProfiler R package (version 3.12.0) (Yu et al., 2012). We selected GO terms with an adjusted *p*-value threshold < 0.05 and considered them to be significantly enriched terms. The balance of kinase and phosphatase activities determines protein phosphorylation status. Specifically, we screened AO targets that regulate kinase activity, phosphatase activity, serine, tyrosine, and threonine phosphorylation and protein autophosphorylation. We also constructed a PPI network of AO targets that are associated with protein phosphorylation that interact directly or indirectly with the tau protein (encoded by MAPT).

### Molecular docking simulations

Molecular docking calculations were performed using the LeDock program^13^ (Wang et al., 2016). Briefly, crystal structures of receptor files were obtained from the RCSB Protein Data Bank (PDB database^14^) and processed by the LePro tool. Binding pocket volumes of receptors were analyzed using the LePro tool. The 3D molecular structures of phytochemicals of AO were produced by CORINA classic^15^. All the parameters were set to default for conformation sampling through a combination of simulated annealing and evolutionary optimization. The docking score (kcal/mol) was calculated through the default scoring function. Generally, docking scores lower than –7 kcal/mol indicate good binding affinity, and scores between –4 and –7 kcal/mol indicate moderate interactions.

## Results

### Main bioactive phytochemicals of *Alpinia oxyphylla* Miquel fruits and their pharmacological properties

**FIGURE 1:**
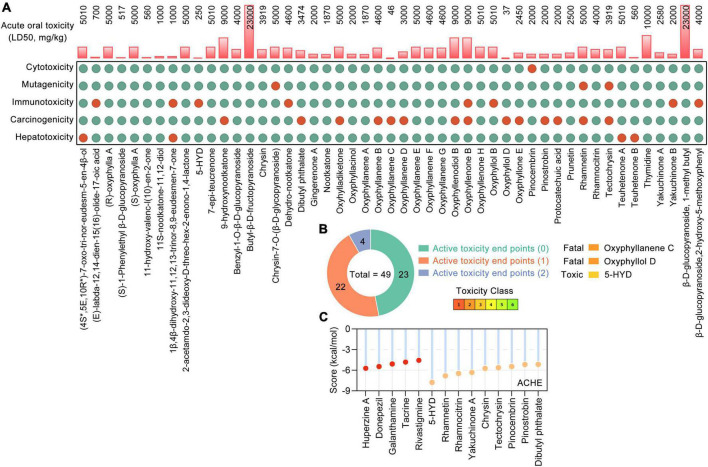
The toxicological parameters of 49 bioactive phytochemicals in *Alpinia oxyphylla* Miq (AO). **(A)** The toxicological parameters including the acute oral toxicity (LD50, mg/kg), hepatotoxicity and toxicological endpoints (carcinogenicity, immunotoxicity, mutagenicity, and cytotoxicity) in AO. Red and green circles represent active or inactive, respectively. **(B)** Numbers of phytochemical with 0 (green), 1 (orange), and 2 (blue) active toxicity endpoints among 49 AO phytochemicals. **(C)** Molecular docking of ACHE with AO phytochemicals and 5 clinically used acetylcholinesterase (ACHE) inhibitors.

Based on the UHPLC-MS/MS data (Xu et al., 2012; Shi et al., 2014; Zhang et al., 2015; Sun et al., 2016; Chen Y. et al., 2020; Ying et al., 2021) and RO5, a total of 49 phytochemicals of AO were shown to meet RO5 and identified as bioactive phytochemicals (Table 1). The main constituents of 49 phytochemicals of AO are sesquiterpenoids (17 constituents, e.g., 7-epi-teucrenone, oxyphyllenone H, oxyphyllone E, oxyphyllol B, and oxyphyllanene A), flavonoids (8 constituents, e.g., chrysin, pinocembrin, pinostrobin, prunetin, rhamnocitrin, and tectochrysin), sesquiterpenes (6 constituents, e.g., 9-hydroxynootkatone, oxyhylladiketone, and nootkatone), and diarylheptanoids (4 constituents, e.g., gingerenone A, oxyphyllacinol, yakuchinone A, and yakuchinone B). All the bioactive phytochemicals of AO have high oral bioavailability, with (R)-oxyphylla A and (S)-oxyphylla A having the highest HOB (79.02), followed by thymidine (78.72), pinocembrin (77.8), and chrysin (75.95) (Table 1). We further evaluated the toxicological parameters of these 49 phytochemicals by using the Protox II web server (Banerjee et al., 2018; Figure 1A). Among these bioactive phytochemicals, 23, 22, and 4 phytochemicals had 0, 1, and 2 active toxicity end points, respectively (Figure 1B). When the toxic compound threshold was set to an acute toxicity lower than Class-3 and having more than three active toxicity end points, oxyphyllanene C, oxyphyllol D, and 5-hydroxy-1,7-bis (4-hydroxy-3-methoxyphenyl) heptan-3-one (5-HYD) were considered to be toxic phytochemicals. The quality marker (Q-marker) is a new paradigm for the quality research of medical plants in TCM. Q-marker analysis based on the components in blood showed that the phytochemical components 7-epi-teucrenone, chrysin, nootkatone, oxyphyllacinol, oxyphyllenone B, protocatechuic acid, tectochrysin, teuhetenone A, and yakuchinone A could be considered Q-markers of AO (Sui et al., 2020). The above results demonstrated that most phytochemicals of AO are RO5-compliant and non-toxic and have high HOB.

**TABLE 1:** Pharmacological and molecular properties of the main phytochemicals in AO.

Compounds	Formula	HOB	MW (g/mol)	Hdon	Hacc	Rbon	LogP
(4S*,5E,10R*)-7-oxo-tri-nor-eudesm-5-en-4β-ol	C_12_H_18_O_2_	59.27	194.27	1	2	0	2
(E)-labda-12,14-dien-15(16)-olide-17-oic acid	C_20_H_28_O_4_	49.97	332.43	1	4	3	3.84
(R)-oxyphylla A	C_12_H_16_O_3_	79.02	208.25	2	3	4	2.22
(S)-1-Phenylethyl β-D-glucopyranoside	C_14_H_20_O_6_	45.18	284.31	4	6	4	–0.06
(S)-oxyphylla A	C_12_H_16_O_3_	79.02	208.25	2	3	4	2.22
11-hydroxy-valenc-l(10)-en-2-one	C_15_H_24_O_2_	45.18	236.35	1	2	1	2.74
11S-nootkatone-11,12-diol	C_15_H_24_O_3_	46.16	252.35	2	3	2	1.95
1β,4β-dihydroxy-11,12,13-trinor-8,9-eudesmen-7-one	C_12_H_18_O_3_	61.43	210.27	2	3	0	1.15
2-acetamdo-2,3-dideoxy-D-threo-hex-2-enono-1,4-lactone	C_8_H_11_NO_5_	71.78	201.18	3	5	4	–0.94
5-Hydroxy-1,7-bis (4-hydroxy-3-methoxyphenyl) heptan-3-one	C_21_H_26_O_6_	35.37	374.43	3	6	10	2.92
7-epi-teucrenone	C_15_H_22_O_2_	49.81	234.33	1	2	1	2.7
9-hydroxynootkatone	C_14_H_20_O_3_	58.73	236.31	1	3	1	1.61
Benzyl-1-*O*-β-D-glucopyranoside	C_13_H_18_O_6_	47.26	270.28	4	6	4	–0.52
Butyl-β-D-fructopyranoside	C_10_H_20_O_6_	57.09	236.26	4	6	5	–0.67
Chrysin	C_15_H_10_O_4_	75.95	254.24	2	4	1	2.55
Chrysin-7-*O*-(β-D-glycopyranoside)	C_22_H_22_O_8_	36.94	414.41	5	8	4	1.38
Dehydro-nootkatone	C_15_H_20_O		216.32	0	1	1	3.41
Dibutyl phthalate	C_16_H_22_O_4_	64.54	278.34	0	4	10	3.69
Gingerenone A	C_21_H_24_O_5_	44.15	356.41	2	5	9	3.65
Nootkatone	C_15_H_22_O	33.04	218.33	0	1	1	3.58
Oxyhylladiketone	C_14_H_20_O_3_	64.21	236.31	1	3	1	2
Oxyphyllacinol	C_20_H_26_O_3_	38.71	314.42	2	3	9	4.12
Oxyphyllanene A	C_12_H_16_O_2_		192.25	0	2	0	1.88
Oxyphyllanene B	C_12_H_14_O_2_		190.24	0	2	0	1.84
Oxyphyllanene C	C_14_H_18_O_3_		234.29	0	3	1	1.95
Oxyphyllanene D	C_15_H_22_O_3_	50.68	250.33	1	3	1	2.38
Oxyphyllanene E	C_15_H_24_O_2_	45.87	236.35	2	2	1	2.66
Oxyphyllanene F	C_15_H_22_O_3_	54.78	250.33	2	3	2	1.91
Oxyphyllanene G	C_15_H_22_O_3_	54.78	250.33	2	3	2	1.94
Oxyphyllenodiol B	C_14_H_22_O_3_	59.8	238.32	2	3	1	1.71
Oxyphyllenone B	C_12_H_18_O_3_	63.64	210.27	2	3	0	1.12
Oxyphyllenone H	C_14_H_22_O_2_	55.78	222.32	1	2	1	2.44
Oxyphyllol B	C_15_H_22_O_2_	43.82	234.33	1	2	1	2.68
Oxyphyllol D	C_15_H_24_O_3_	42.7	252.35	2	3	0	2.01
Oxyphyllone E	C_14_H_20_O_2_		220.31	0	2	1	2.45
Pinocembrin	C_15_H_12_O_4_	77.8	256.25	2	4	1	2.26
Pinostrobin	C_16_H_14_O_4_	71.57	270.28	1	4	2	2.66
Protocatechuic acid	C_7_H_6_O_4_	49.54	154.12	3	4	1	0.65
Prunetin	C_16_H_12_O_5_	64.68	284.26	2	5	2	2.43
Rhamnetin	C_16_H_12_O_7_	42.07	316.26	4	7	2	1.63
Rhamnocitrin	C_16_H_12_O_6_	52.79	300.26	3	6	2	1.98
Tectochrysin	C_16_H_12_O_4_	71.46	268.26	1	4	2	2.95
Teuhetenone A	C_12_H_18_O_2_	59.27	194.27	1	2	0	1.98
Teuhetenone B	C_14_H_22_O_2_	48.32	222.32	1	2	1	2.58
Thymidine	C_10_H_14_N_2_O_5_	78.72	242.23	3	5	2	–0.61
Yakuchinone A	C_20_H_24_O_3_	40.12	312.4	1	3	9	4.09
Yakuchinone B	C_20_H_22_O_3_	37.95	310.39	1	3	8	4.15
β-D-glucopyranoside, 1-methyl butyl	C_11_H_22_O_6_	59.29	250.29	4	6	5	–0.37
β-D-glucopyranoside,2-hydroxy-5-methoxyphenyl	C_13_H_18_O_8_	31.05	302.28	5	8	4	–0.78

HOB, human oral bioavailability; MW, molecule weight; Hdon, number of hydrogen bond donors; Hacc, number of hydrogen bond acceptors; Rbon, number of rotatable bonds; LogP, lipid-water partition coefficient. *Stands for highlighting.

According to the SwissTargetPrediction web server (Daina et al., 2019) and the structures of 49 bioactive phytochemicals of AO, 662 potential targets of AO, including ACHE and BCHE, were identified. Nine phytochemicals of AO were shown to target ACHE, and 19 phytochemicals were shown to target BCHE. Chrysin, pinocembrin, pinostrobin, rhamnocitrin, and tectochrysin target both ACHE and BCHE. By molecular docking, we analyzed the ACHE-binding affinities of five clinically used ACHE inhibitors that are approved by the American Food and Drug Administration (FDA) and phytochemicals of AO. The binding affinities of 5-HYD, rhamnetin, rhamnocitrin, yakuchinone A, and chrysin to AHCE were greater than those of all five clinically used ACHE inhibitors, namely, huperzine, donepezil, galanthamine, tacrine, and rivastigmine (Figure 1C). Tectochrysin, pinocembrin, pinostrobin, and dibutyl phthalate also have similar ACHE-binding affinities as those five ACHE inhibitors (Figure 1C). These results indicate that AO could improve ACh levels by inhibiting ACh hydrolysis.

### The gene basis of dementia with kidney-essence deficiency and the bioactive phytochemicals of *Alpinia oxyphylla* Miquel that function in tonifying kidney-essence and nourishing intelligence

**FIGURE 2:**
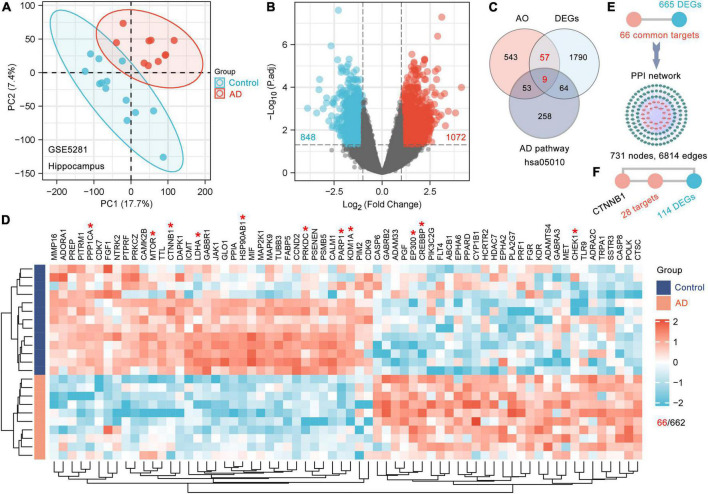
The gene basis for the cognitive impairment in kidney deficiency. **(A)** PCA displays sample clustering within age-matched control (*n* = 13) and AD hippocampal brain samples (*n* = 10). **(B)** Volcano plot of DEGs between the AD and control brain. The threshold parameters used were | Log_2_ (fold change)| > 1 and *p* adjust < 0.05. **(C)** Venn diagram showing the shared genes among AO targets, DEGs and AD pathway (hsa05010). **(D)** Clustering heatmap of 66 AO targets differentially expressed in AD hippocampus. Red and green represent high and low expressed genes, respectively. The Top 10 AO-regulated DEGs ranked by degree with an asterisk in the heatmap were marked red **(D)**. **(E)** 665 DEGs directly interact with 66 AO-regulated DEGs as shown in the PPI network. **(F)** CTNNB1 (β-catenin) directly interacts with 28 AO-regulated DEGs.

To date, there have been no reports about the biological basis of the induction of dementia by kidney-essence deficiency. AD is a typical disease with deficient kidney-essence in TCM. Therefore, we tried to identify the genes that are responsive to AO among the DEGs between AD patients and age-matched controls and considered these DEGs to be the biological basis of dementia with kidney-essence deficiency. The GSE5281 dataset (Liang et al., 2007) was obtained from the GEO database. The 23 samples from the human hippocampus included 13 control samples (age: 79.6 ± 2.6 years) and 10 AD samples (age: 77.8 ± 1.8 years). PCA showed that samples were scattered among groups and clustered within groups, indicating good duplication within groups and significant differences between groups (Figure 2A). A total of 1920 DEGs (1072 upregulated and 848 downregulated) were obtained after screening genes with the criteria of fold change > 2 and *p*-adjusted < 0.05 (Figure 2B), and 66 DEGs were AO targets (Figures 2C,D). Furthermore, we used 66 AO-targeted DEGs as the seed nodes to construct a PPI network; we found that 665 DEGs directly interacted with these nodes and formed a PPI network with 731 nodes and 6814 edges (Figure 2E). Therefore, we considered these 731 DEGs to be the gene basis for dementia with kidney-essence deficiency. CHEK1, CREBBP, CTNNB1 (β-catenin), EP300, HSP90AB1, KDM1A, LDHA, MTOR, PARP1, PPP1CA, and PRKDC had the highest levels of interaction (Figure 2D, indicated by red asterisks). CTNNB1, which was one of the top 10 most frequently interacting molecules, was shown to interact with 142 DEGs, including 28 DEGs that were directly regulated by AO (Figure 2F). This suggests that CTNNB1 is an important target gene of AO in the treatment of cognitive impairment in kidney deficiency.

**FIGURE 3:**
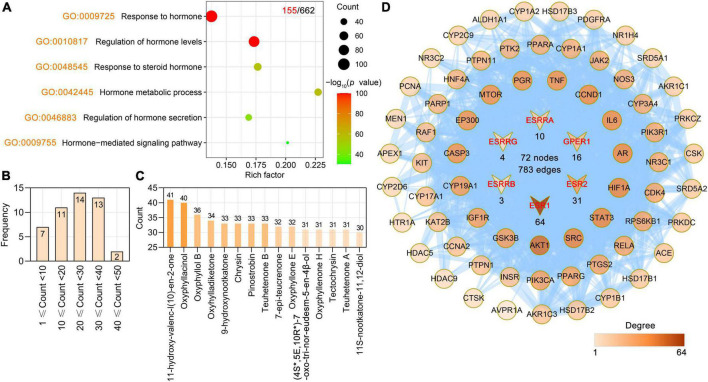
*Alpinia oxyphylla* Miquel (AO) regulates hormone-related targets and their phytochemical components. **(A)** Bubble plot showing regulatory hormone-related BP terms in AO targets. The *x*-axis, rich factor (the ratio between the number of targets and the number of the background genes). Bubble size, the number of genes enriched. Bubble color, –Log_10_ (*p*-value). **(B)** Frequency distribution of AO phytochemicals corresponding to 155 hormone-related targets. **(C)** Number of AO phytochemical targets targeting 155 hormone-related targets. **(D)** PPI network reveals 66 AO targets interact directly with 6 estrogen-related receptors (ESR1, ESR2, ESRRA, ESRRB, ESRRG, GPER1).

Hormone may be a key player in cognitive dysfunction and AD pathology (Choi et al., 2020; Nature, 2022). For example, estrogen affects the brain in several different ways, some of which researchers think could help explain how it could protect against AD. Estrogen can also affect the way chemicals such as serotonin, acetylcholine, and dopamine are used to send signals throughout the brain. Some of the symptoms of Alzheimer’s disease are linked to problems with the acetylcholine signaling system, which could be connected to decreased estrogen levels. We found that the targets of AO were involved in biological process (BP) terms related to hormones. By ClusterProfiler R package-based GO analysis, the primary enriched hormone-related BP terms were response to hormone (GO: 0009725), regulation of hormone levels (GO: 0010817), response to steroid hormone (GO: 0048545), hormone metabolic process (GO: 0042445), regulation of hormone secretion (GO: 0046883), and hormone-mediated signaling pathway (GO: 0009755) (Figure 3A). Further study showed that 47 phytochemicals of AO had 155 hormone-related targets, and 15 phytochemicals of AO had more than 30 hormone-related targets (Figure 3B). 11-Hydroxy-valenc-l (10)-en-2-one, oxyphyllacinol, oxyphyllol B, oxyhylladiketone, 9-hydroxynootkatone, chrysin, pinostrobin, and teuhetenone B had the highest numbers of hormone-related targets (Figure 3C). Among these 155 targets, 66 AO targets directly interacted with six estrogen receptors (ERs), e.g., ESR1, ESR2, ESRRA, ESRRB, ESRRG, and GPER1 (Figure 3D).

**FIGURE 4:**
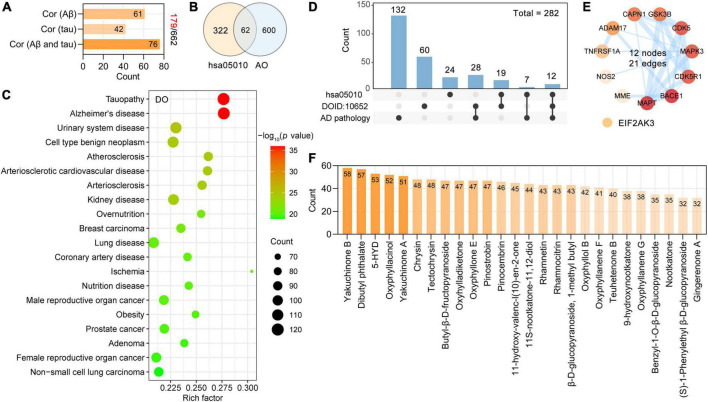
Screening potential targets of AO against AD and its corresponding phytochemicals. **(A)** Number of AO targets correlated with Aβ pathology, tau pathology, Aβ and tau pathology, respectively. **(B)** Venn diagram showing overlapping targets between the Alzheimer’s disease (hsa05010) and AO targets. **(C)** Top 20 significantly enriched Disease Ontology (DO) terms are shown as a bubble diagram. DO terms with corrected *p*-value < 0.05 were considered significantly enriched. *X*-axis, rich factor (the ratio of targets in the background terms). Bubble size, the number of genes enriched. Bubble color, –Log_10_ (*p*-value). **(D)** UpSetR plot highlights the intersection of AO targets associated with AD pathology, Alzheimer’s disease (hsa05010) and Alzheimer’s disease (DOID: 10652). **(E)** PPI network of 12 anti-AD targets related to AD pathology, Alzheimer’s disease (hsa05010) and Alzheimer’s disease (DOID: 10652). The thickness of the edges represents the combined score. **(F)** Phytochemicals of AO with more than 30 anti-AD targets.

As described, we used three steps to identify the neurodegenerative dementia-related targets among the AO targets. First, we found that 27% of AO targets (179 targets) were significantly associated with Aβ and tau pathology, which are key neuropathological features of neurodegenerative dementia. There were 61, 42, and 76 AO targets that were closely associated with Aβ pathology, tau pathology, and Aβ and tau pathology, respectively (Figure 4A). Second, we identified 62 of the 384 targets in the Alzheimer’s disease pathway (hsa05010) as AO targets (Figure 4B), and these targets are involved in the production and clearance of Aβ, tau phosphorylation and neuron death. Third, we performed DO enrichment analysis to determine the relevance of these AO targets to human disease. A total of 602 DO terms were significantly enriched in our study, and DO terms with an adjusted *p*-value < 0.05 were considered to be significantly enriched.

As shown, the primary enriched DO terms were tauopathy (DOID: 680), Alzheimer’s disease (DOID: 10652), urinary system disease (DOID: 18), cell type benign neoplasm (DOID: 0060084), and atherosclerosis (DOID: 1936) (Figure 4C). AD was significantly enriched (adjust *p*-value = 1.13E-36), and 119 targets were involved in this term. Pooled targets of AO in the treatment of AD are summarized in Figure 4D, and 282 targets of AO in AD treatment were ultimately identified. The details of these 282 targets are provided in Supplementary Table 1. AlzData was used to analyze the relationship between these 282 targets and cell types (astrocytes, microglia, and neurons) in the human brain, and ultimately, 159 targets were classified. Thirty-four targets were specifically localized to neurons, 23 targets to microglia, and 8 targets to astrocytes, indicating that AO may act on these major types of cells in the brain (Supplementary Figure 1). As shown in Figure 4D, 12 AO targets, ADAM17, BACE1, CAPN1, cyclin-dependent kinase 5 (CDK5), CDK5R1, GSK3B, MAPT, MME, and NOS2, were identified by all three screening strategies and formed a PPI network with 12 nodes and 21 edges (Figure 4E). By studying the phytochemicals corresponding to 282 targets of AO in the treatment of AD, we found that yakuchinone B (58 targets), dibutyl phthalate (57 targets), 5-HYD (53 targets), oxyphyllacinol (52 targets), yakuchinone A (51 targets), chrysin (48 targets), and tectochrysin (48 targets) had the highest number of targets in the treatment of AD (Figure 4F). Figure 4F shows 26 phytochemicals of AO with more than 30 anti-AD targets (Supplementary Table 2).

### Key phytochemicals of *Alpinia oxyphylla* Miquel that are associated with Aβ deposition

Cerebral Aβ deposition is an important pathophysiological event in aging-related dementia. Aβ exerts neurotoxic effects that impair the BBB, induce losses of neurons and synapses, trigger neuroinflammation, and damage synaptic transmission (Steinman et al., 2020; Wang et al., 2021; Karran and De Strooper, 2022; Li and Stern, 2022). Vascular Aβ deposition-induced cerebral amyloid angiopathy (CAA) is a risk factor for dementia (Jakel et al., 2017; Abubakar et al., 2022). Aβ deposition inside and outside of neurons is a typical feature of AD (Tikhonova et al., 2021). Therefore, reducing the Aβ load in the brain has become an important strategy for the treatment of related diseases.

**FIGURE 5:**
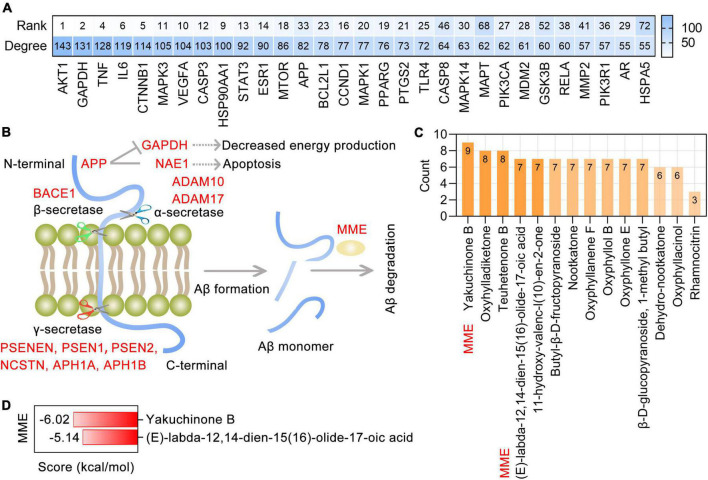
Targeting AO targets associated with Aβ production and degradation. **(A)** Top 30 core targets of AO’s 282 anti-AD targets were ranked by degree, and the heatmap also shows the ranking of anti-AD core targets in the PPI network formed by all 662 targets of AO. **(B)** Schematic diagram of the Aβ production and Aβ degradation, with AO targets shown in red font. Aβ is produced by α-, β-, and γ-secretase-mediated cleavage of APP and efficiently degraded by MME. **(C)** AO phytochemicals involved in Aβ production and degradation ranked according to the numbers of targets. **(D)** Molecular docking of the yakuchinone B, (E)-labda-12,14-dien-15(16)-olide-17-oic acid with MME, which is one of the most prominent Aβ-degrading enzymes.

Protein-protein interaction analysis showed a remarkably significant enrichment of known interactions among 282 targets of AO in AD treatment (PPI enrichment *p*-value < 1.0E-16). The resulting PPI network contained 280 nodes and 3588 edges, and the top 30 targets ranked by degree were identified as core targets (Figure 5A). Notably, these core targets, especially AKT1, GAPDH, TNF, IL6, CTNNB1, MAPK3, VEGFA, CASP3, HSP90AA1, STAT3, ESR1, and MTOR, were highly ranked in the PPI network formed by all 662 AO targets. This suggests that AO may be the herb that is most relevant to AD treatment. APP is a transmembrane protein that is widely distributed in the brain and peripheral organs, and in the human brain, it is primarily localized to neurons. The production of Aβ depends on the cleavage of APP by α-secretase (ADAM10 and ADAM17), β-secretases (BACE1 and BACE2) and γ-secretases (APH1A, APH1B, NCSTN, PSEN1, PSEN2, and PSENEN). MME (encodes neprilysin) and IDE (insulin degrading enzyme) are the most prominent Aβ-degrading enzymes (Figure 5B). Based on the 13 Aβ production- and degradation-related targets of AO shown in Figure 5C, the corresponding phytochemicals were identified. There were 14 phytochemicals of AO with more than 3 targets involved in Aβ production and degradation (Figure 5C). Among these phytochemicals, (*E*)-labda-12,14-dien-15(16)-olide-17-oic acid and yakuchinone B have significant binding affinity for MME and participate in the degradation of Aβ (Figures 5C,D). Therefore, the therapeutic targeting of the production and degradation of Aβ may be one of the important mechanisms by which AO function in the treatment and prevention of AD.

### Major effects of the bioactive phytochemicals of *Alpinia oxyphylla* Miquel on tau aggregation

**FIGURE 6:**
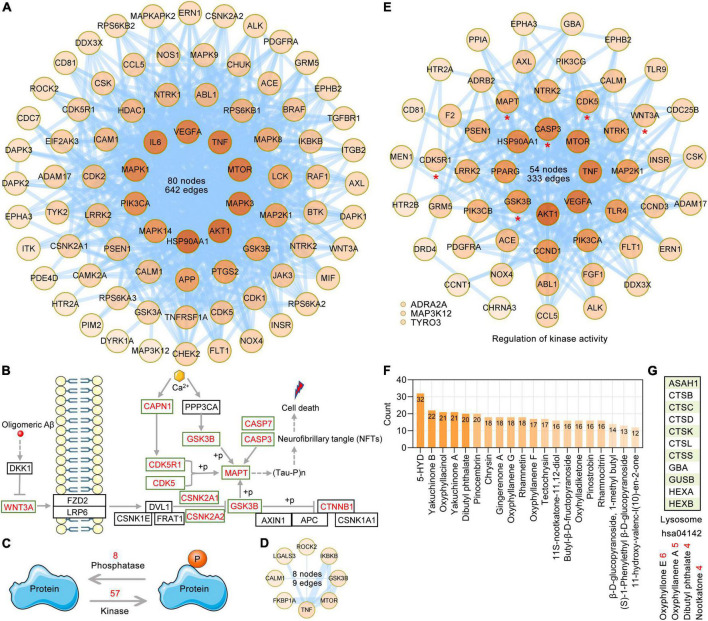
Targeting AO targets associated with tau phosphorylation and degradation. **(A)** The PPI network was constructed for the 83 potential targets of AO associated with protein phosphorylation. Node color alludes to the degree value. **(B)** Schematic diagram of the regulatory mechanism of tau phosphorylation. The red font targets represent the AO targets involved in the regulation of tau phosphorylation. **(C)** The balance between protein kinase and phosphatase activities regulates the phosphorylation of protein. **(D)** PPI network of 8 AO targets involved in regulation of phosphatase activity (GO: 0010921). The thickness of the edges is correlated with the combined score. **(E)** PPI network construction for 57 AO targets involved in regulation of kinase activity (GO: 0033673 and GO: 0033674). **(F)** AO phytochemicals involved in tau phosphorylation ranked according to the numbers of targets. **(G)** AO phytochemicals corresponding to targets involved in lysosome pathway (hsa04142).

Tau is a microtubule-associated protein. Tauopathies are a heterogeneous group of neurodegenerative dementias (including AD) that are characterized by the formation of tau aggresomes, such as NFTs (Almansoub et al., 2019; Mroczko et al., 2019; Papanikolopoulou and Skoulakis, 2020). Independent of the aggregation and destabilization of microtubules, phosphorylated tau increases Aβ toxicity and leads to the impairment of synaptic function and memory formation in a mouse model of AD. Furthermore, BBB integrity and functionality may be impacted by pathological tau (Canepa and Fossati, 2020). The synthesis, folding, post-translational modification and degradation of tau affect cellular homeostasis. There are various post-translational modifications of tau, such as phosphorylation, glycosylation, ubiquitination, and glycation; among these modifications, phosphorylation, which is the most well-studied, is mainly caused by the deregulation of kinases and phosphatases. According to the GO enrichment analysis of 282 AO targets in the treatment of AD, GO BP terms related to protein phosphorylation regulation, such as peptidyl-serine phosphorylation (GO: 0018105), peptidyl-threonine phosphorylation (GO: 0018107), peptidyl-tyrosine phosphorylation (GO: 0018108), and protein autophosphorylation (GO: 0046777), were significantly enriched, and a total of 83 AO targets were identified. PPI analysis showed that the PPI network formed by these 83 AO targets contained 80 nodes and 642 edges (Figure 6A). According to the Alzheimer’s disease pathway (hsa05010), we listed the main AO targets that regulate tau phosphorylation, such as MAPT, GSK3B, CDK5, CDK5R1, and CAPN1 (Figure 6B). The phosphorylation status of a protein is the result of the balance between kinase and phosphatase activities. Based on BP terms (GO: 0010921, regulation of phosphatase activity; GO: 0033673, negative regulation of kinase activity; GO: 0033674, positive regulation of kinase activity), we identified 8 and 57 AO targets that regulate kinase and phosphatase activities, respectively (Figure 6C). PPI analysis showed that 8 AO targets that regulate phosphatase activity included 8 nodes and 9 edges (Figure 6D), while 57 AO targets that regulate kinase activity included 54 nodes and 333 edges (Figure 6E). When these AO targets that regulate protein phosphorylation were pooled, a total of 105 targets were identified. PPI analysis revealed that 33 of 105 AO targets that regulate protein phosphorylation had direct interactions with MAPT, and 66 targets indirectly interacted with MAPT (1 node interval) (Supplementary Figure 2). Based on these data, we also refer to these targets that regulate protein phosphorylation as targets that regulate tau phosphorylation. Importantly, 18 AO targets that regulate tau phosphorylation are the core targets of AO in the treatment of AD, including AKT1, APP, CASP3, CCND1, GSK3B, MAPK1, MAPK3, and MAPT (Figure 5A). In Figure 6F, we list 20 phytochemicals of AO that regulate more than 12 tau phosphorylation targets. 5-HYD, yakuchinone B, oxyphyllacinol, yakuchinone A, dibutyl phthalate and pinocembrin were the top six regulators of tau hyperphosphorylation. The degradation of tau mainly occurs *via* the ubiquitin-proteasome pathway and the lysosomal proteolysis pathway, and inhibition of these pathways promote tau aggregation (Wang et al., 2009). KEGG enrichment analysis revealed that 11 AO targets were significantly enriched in the lysosome pathway (hsa04142, *p* = 0.008), but not the proteasome pathway (Figure 6G). The possible targets that regulate tau lysosomal degradation are suggested to be oxyphyllone E, oxyphyllanene A, dibutyl phthalate, and nootkatone.

### *Alpinia oxyphylla* Miquel targets and their phytochemicals that regulate neuroinflammation

**FIGURE 7:**
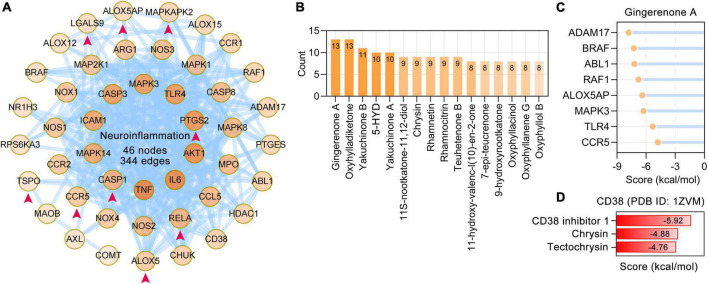
*Alpinia oxyphylla* Miquel (AO) targets and their phytochemicals targeting neuroinflammation. **(A)** The PPI network was constructed for the 47 targets of AO associated with neuroinflammation. AO targets specifically localized to microglia were pointed out by red arrows. The darker the color, the higher the degree. **(B)** AO phytochemicals involved in neuroinflammation ranked according to the numbers of targets. **(C)** Molecular docking of gingerenone A with proteins involved in neuroinflammation. **(D)** Molecular docking of the tectochrysin, chrysin and CD38 inhibitor 1 (compound 78c) with CD38.

Neuroinflammation is an important aspect of the pathogenesis of neurodegenerative dementia (Heneka et al., 2015). Based on PubMed searches and enriched BP terms, 47 neuroinflammation-related AO targets were identified and formed a PPI network with 46 nodes and 344 edges. The AO targets 5-lipoxygenase (ALOX5), ALOX5AP, CASP1, CCR5, LGALS9, MAPKAPK2, PTGS2, RELA, and TSPO that are involved in neuroinflammation were specifically enriched in microglia (Figure 7A). The possible regulators of neuroinflammation were suggested by the number of related targets (Figure 7B). Gingerenone A (13 targets), oxyhylladiketone (13 targets), yakuchinone B (11 targets), 5-HYD (10 targets), and yakuchinone A (10 targets) were the top 5 regulators of neuroinflammation. Molecular docking results revealed that gingerenone A had stable interactions with ADAM17, BRAF, ABL1, RAF1, ALOX5AP, MAPK3, TLR4, and CCR5 (Figure 7C). CD38 is important in both neurodegeneration and neuroinflammation (Guerreiro et al., 2020). Molecular docking revealed that chrysin and tectochrysin have high binding affinity for CD38, similar to the endogenous inhibitor of CD38 (CD38 inhibitor 1) (Figure 7D).

### Major effects of bioactive phytochemicals of *Alpinia oxyphylla* Miquel on neuron death

**FIGURE 8:**
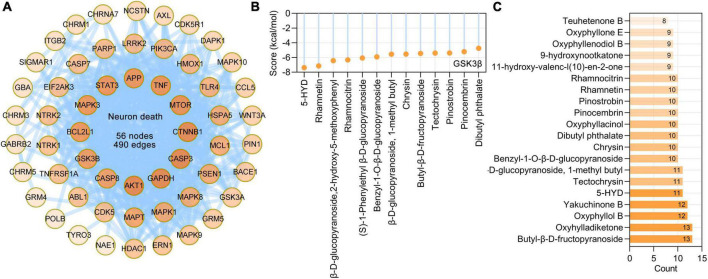
*Alpinia oxyphylla* Miquel (AO) targets and their phytochemicals targeting neuron death. **(A)** The PPI network of AO targets related to neuron death was constructed by STRING database. **(B)** Number of AO phytochemical targets targeting neuron death. **(C)** Molecular docking of the GSK3β (PDB ID: 1J1C) with its corresponding AO phytochemicals. Docking scores provided by Ledock represent predicted binding energies in kcal/mol.

Loss of neurons is one pathological hallmark of neurodegenerative dementia. Based on BP terms (GO: 1901214, regulation of neuron death) and calcium-induced neuronal death in Alzheimer’s disease pathway (hsa05010), we identified 56 AO targets that are involved in neuron death, and these targets formed a PPI network with 56 nodes and 490 edges (Figure 8A). Further analysis showed that the main phytochemicals that target these molecules are butyl-β-D-fructopyranoside (13 targets), oxyhylladiketone (13 targets), oxyphyllol B (12 targets), yakuchinone B (12 targets), 5-HYD (11 targets), tectochrysin and β-D-glucopyranoside (11 targets), and 1-methyl butyl (11 targets) (Figure 8B). GSK3β (encoded by GSK3B) has been demonstrated to be important in neuronal death (Arciniegas and Eldar-Finkelman, 2021). Molecular docking results showed that 5-HYD, rhamnetin, β-D-glucopyranoside, 2-hydroxy-5-methoxyphenyl, rhamnocitrin, and (S)-1-phenylethyl β-D-glucopyranoside had strong binding affinities for GSK3β (Figure 8C).

**FIGURE 9:**
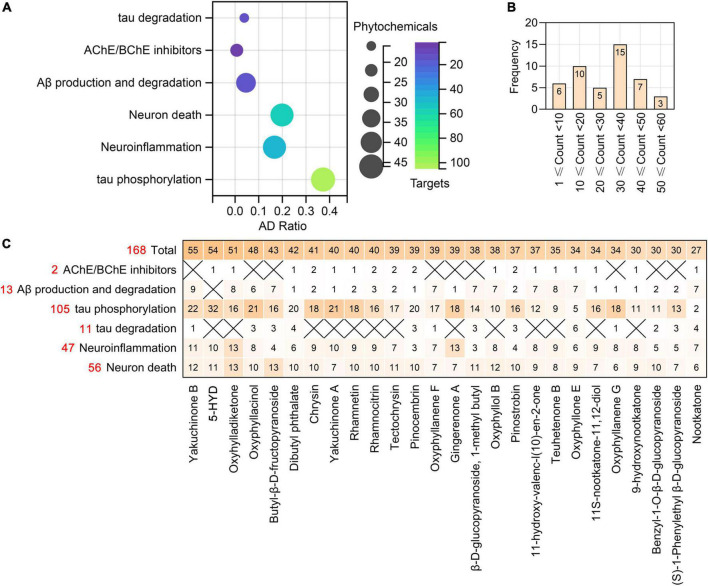
The phytochemicals and targets of AO in treating neurodegenerative dementia. **(A)** Bubble plot showing the number of phytochemicals of AO targeting tau degradation, ACHE/BCHE, Aβ production and degradation, tau phosphorylation, neuroinflammation, and neuron death. The *x*-axis, AD Ratio (the ratio of the targets in the total targets). Bubble size, the number of AO phytochemicals. Bubble color, the number of targets. **(B)** Frequency distribution of AO phytochemicals with different numbers of anti-AD targets. **(C)** Heatmap summarizes the number of targets for AO phytochemicals regulating different pathological processes. *X* indicates not applicable. The red numbers represent the total number of targets involved in each row in the heatmap.

According to all the results presented above, we summarize the targets of the phytochemicals of AO that regulate anticholinesterase activity, Aβ production and degradation, tau phosphorylation, tau degradation, neuroinflammation and neuron death (Figure 9 and Supplementary Table 3). There were 16, 23, 37, 44, 44, and 46 phytochemicals of AO that target molecules related to tau degradation, anticholinesterase activity, Aβ production and degradation, neuroinflammation, neuron death, and tau phosphorylation, respectively (Figure 9A). According to Figure 9B, there are 25 phytochemicals of AO with more than 30 targets related to pathological processes of AD. We show not only the total number of targets of phytochemicals of AO in the treatment of AD but also the number of targets of phytochemicals of AO that are relevant to each pathological feature of AD (Figure 9C). The chemical structures of phytochemicals of AO that target the top 26 molecules in the treatment of AD are presented in Supplementary Figure 3. It is worth noting that 2, 18, 11, and 17 AO targets that regulate Aβ production and degradation, tau phosphorylation, neuroinflammation, and neuron death are core targets in the treatment of AD (Supplementary Figure 4).

## Discussion

**FIGURE 10:**
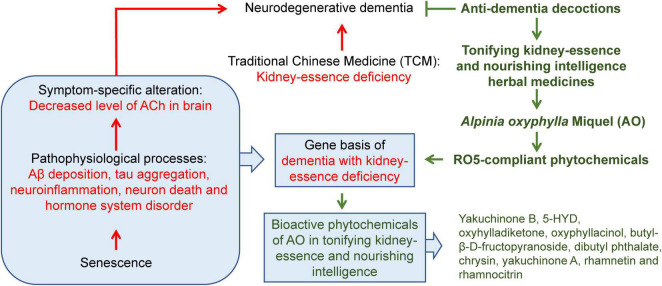
A schematic diagram of the overall conclusion of this study.

The current efficacy of clinical neurodegenerative dementia treatment is limited, as the commonly used drugs target symptoms and do not affect key pathophysiological processes. Additionally, there are several failures in terms of developing disease-modifying therapies based on single pathological changes (Mangialasche et al., 2010). Therefore, the development of drugs that target multiple pathophysiological processes is very promising. To fully understand the pharmacological effects of AO in the treatment of neurodegenerative dementia, we focused not only on the metabolism of ACh but also on five important pathophysiological processes, e.g., Aβ deposition, tau aggregation, neuroinflammation, neuron death, and hormone system disorder. A schematic diagram of the overall conclusion of this study is presented in Figure 10.

In comparison to previous studies (Xu et al., 2020; Li W. J. et al., 2021), the multilevel pathophysiological process-based methodology of this study has some advances. First, multilevel pathophysiological process data provide a more comprehensive and systematic view of underlying mechanisms. Based on comprehensive evaluations, we prioritized 26 phytochemicals of AO that target multiple pathophysiological processes (Figure 9). Second, the results of this study are highly robust and reliable. The phytochemicals of AO examined in this study were identified by UHPLC-MS/MS studies (Xu et al., 2012; Shi et al., 2014; Zhang et al., 2015; Sun et al., 2016; Chen Y. et al., 2020; Ying et al., 2021), and objective methods were used to identify AO targets the treatment of AD. It is worth noting that the bioactive phytochemicals of AO examined in this study are highly specific. For example, oxyphyllol B, yakuchinone A, oxyphyllacinol, tectochrysin, and yakuchinone B are present only in 1, 2, 4, 5, and 5 herbs, respectively, including AO (data from HERB^16^) (Fang et al., 2021). In general, widely distributed phytochemicals do not account for the specific pharmacological effects of any particular herb. Third, the both pharmacological and toxicological parameters of the phytochemicals of AO were evaluated in this study. Among 49 phytochemicals of AO, oxyphyllanene C, oxyphyllol D, and 5-HYD were shown to be potentially toxic. 5-HYD, the fourth most abundant chemical component of AO (Ying et al., 2021), has the second largest number of targets in AD treatment, and it targets several different pathological processes related to AD. Although 5-HYD exhibits excellent anti-AD activity, special attention should be given to its potential toxicity in follow-up studies.

We acknowledge some limitations of this study. Not all the phytochemicals of AO have been verified in animal or cell models of dementia in previous studies. Therefore, these phytochemicals must be validated in various animal models of neurodegenerative dementia and in clinical trials before they can be used in patients. This study considered only the target proteins that correspond to phytochemicals and did not consider other biological function molecules.

ACHE and BCHE co-regulate the metabolism of the neurotransmitter ACh, and ACHE inhibitors have been developed for the symptomatic treatment of AD. *In vitro* experiments showed that the ethanol extract of AO had strong ACHE inhibitory activity, and the inhibitory activity at the final concentration of 0.1 mg/mL was 44.49 ± 3.66% (Chen et al., 2013). In a scopolamine-induced cognitive impairment mouse model of AD, the oral administration of AO for 30 days increased the levels of ACh and M1 receptors and decreased the activity of ACHE (Wang M. et al., 2018). The AHCE-binding affinities of 5-HYD, rhamnetin, rhamnocitrin, yakuchinone A, and chrysin are greater than those of the currently known FDA-approved ACHE inhibitors. Chrysin and rhamnocitrin also target BCHE. Previous studies have shown that nootkatone is a cholinesterase inhibitor of ACHE and BCHE (Sanchez-Martinez et al., 2021). Intracerebroventricular injection of nootkatone (0.02 mg/kg and 0.2 mg/kg) ameliorates Aβ_1–42_-induced cognitive impairment by upregulating antioxidant and anti-ACHE activities in the hippocampus (He et al., 2018). A recent study showed that chrysin derivatives are potent inhibitors of ACHE and BCHE and also inhibit Aβ_1–42_ aggregation (Yang A. et al., 2022). The phytochemicals of these potential ACHE and BCHE inhibitors need to be further explored in future work.

Aβ deposition and tau pathology predate structural changes in the brain, including reduced hippocampal volume and decreased glucose metabolism, by decades (Jack et al., 1999). Since the imbalance between Aβ production and clearance is often considered an early initiation factor, reducing Aβ production and/or increasing Aβ clearance are key therapeutic strategies for AD treatment. The pathogenesis of Aβ begins with the alteration of APP, which is cleaved by α-secretase (ADAM10, ADAM17), BACE1, and γ-secretase (APH1A, APH1B, NCSTN, PSEN1, PSEN2, PSENEN). APP is generally believed to exert positive effects on cell health and growth (Oh et al., 2009). Interestingly, APP and the secretases that cleave APP are widely expressed in many tissues, including the brain (Roher et al., 2009). This suggests that peripherally produced Aβ is one of the many sources of total Aβ, and the clearance of peripheral Aβ is also a promising strategy for AD treatment. A previous study also demonstrated a significant contribution of the peripheral system to Aβ clearance in the brain (Xiang et al., 2015). In this study, we found that various phytochemicals of AO might treat AD in a synergistic manner by affecting the production and clearance of Aβ. In an Aβ_1–42_-induced AD mice model, intraventricular injection of tectochrysin (140 μg/kg) for 5 days downregulated β-secretase expression and Aβ_1–42_ accumulation in the brain (He et al., 2019). MME is considered to be the most important Aβ-degrading enzyme (Miners et al., 2012). In this study, (E)-labda-12,14-dien-15(16)-olide-17-oic acid and yakuchinone B were found to target MME and might participate in the degradation of Aβ. Another important Aβ-degrading enzyme, IDE, is not a target of AO. Our analysis revealed that BACE1 is also involved in neuron death. Genetic deletion of BACE1 in 5xFAD mice not only abolished Aβ production and prevented amyloid deposition but also prevented neuron loss in the cerebral cortex and subiculum (Ohno et al., 2007). RO5-compliant compounds have strong abilities to cross the BBB (Mohd et al., 2020). As expected, most of the phytochemicals of AO that target Aβ production and degradation are mostly accessible to the central nervous system by crossing the BBB^17^. A sesquiterpenoid, nootkatone, has shown a strong capacity to cross the BBB *in vitro* (Sanchez-Martinez et al., 2021). Pinocembrin showed great neuroprotective potential in preclinical research with high bioavailability and BBB permeability (Shen et al., 2019; Xu et al., 2021). Therefore, we believe that AO affects Aβ pathology both centrally and peripherally.

The functions of tau are regulated by multiple post-translational modifications at more than 50 sites, and phosphorylation is the most documented (Wegmann et al., 2021). In the frontal and parietal cortex of patients with AD, 43–55 distinct phosphorylation, 19 acetylation, 14–17 ubiquitination, and 4 methylation sites of tau have been identified (Wesseling et al., 2020). Of the 85 putative tau phosphorylation sites, more than 50 sites (serine, threonine, tyrosine) have been confirmed to be modified in tau (Mair et al., 2016). In this study, we found that AO regulates 105 tau phosphorylation-related targets, including multiple kinases and phosphatases. GSK3β and CDK5 are the most relevant kinases that are involved in tau phosphorylation. Mammalian GSK3 consists of the highly homologous GSK3α and GSK3β. GSK3α is particularly abundant in the hippocampus, cerebral cortex, striatum and cerebellum, and GSK3β is expressed in almost all regions of the brain (Woodgett, 1990; Pandey et al., 2009). GSK3 regulates more than 40 putative tau phosphorylation sites, of which at least 29 are hyperphosphorylated in the brains of patients with AD (Hanger et al., 2009). β-catenin is an important downstream target of GSK3α/β. Wnt/β-catenin signaling regulates multiple aspects of AD pathogenesis, including synaptic plasticity, neuronal survival, neurogenesis, BBB integrity, tau phosphorylation, and Aβ production (Jia et al., 2019). In this study, β-catenin was not only the core target of all AO targets but also the most important target of AO in the treatment of AD. Among 13 phytochemicals of AO that bind to GSK3β, chrysin can also target Akt and MAPKs to affect protein phosphorylation (Liu et al., 2016; Nagavally et al., 2021). Pinocembrin, which is a flavonoid, exerts neuroprotective effects through the GSK3β and Akt signaling pathways (Kim et al., 2020). In neurodegenerative diseases such as AD, the activation of GSK3α not only participates in the process of APP hydrolysis to form Aβ but also regulates the phosphorylation of tau (Silva-Garcia et al., 2020). Moreover, GSK3α is important in the regulation of synaptic plasticity. CDK5 regulates microtubule stability, synaptic plasticity, and neuronal cell cycle events in the brain (Allnutt et al., 2020). A total of 16 phytochemicals of AO, including chrysin, oxyhylladiketone, oxyphyllanene D, pinocembrin, yakuchinone A, yakuchinone B, etc., target CDK5 and its receptor CDK5R1. We noticed that GSK3α and GSK3β can regulate not only tau phosphorylation but also neuronal death. Therefore, the corresponding phytochemicals of AO that target tau-related kinases and phosphatases, especially GSK3α, GSK3β and CDK5, should attract more attention.

Inflammation is associated with aging-related diseases, and TCM treatment that tonifies kidney-essence can significantly inhibit inflammation (Jian-Jun et al., 2015). The main cell types involved in the inflammatory response in the brain are microglia and astrocytes. In this study, the targets of AO that are involved in neuroinflammation, e.g., ALOX5, ALOX5AP, CASP1, CCR5, LGALS9, MAPKAPK2, PTGS2, RELA, and TSPO, were specifically enriched in microglia. ALOX5 is an enzyme that is involved in arachidonic acid metabolism, and the ALOX5 pathway may serve as a potential therapeutic target for AD (Chen F. et al., 2020). In AD animal models and brains of AD patients, ALOX5 expression is elevated and involved in regulating AD pathogenesis. A previous study also suggested that ALOX5 and ALOX5AP polymorphisms may be associated with AD (Manev and Manev, 2006). In this study, among the AO targets in the treatment of AD, the chemokine receptor family members CCR1, CCR2, and CCR5 (CCL5) were found to be involved in the regulation of neuroinflammation. CCR1, which is part of the neuroimmune response to Aβ_42_-positive neuritic plaques, is an early specific marker of AD (Halks-Miller et al., 2003). CCR3-, CCR5-, and CXCR2-positive reactive microglia are associated with amyloid deposition in AD. *In vitro*, CCL5 induces pro-inflammatory profile of microglia (Skuljec et al., 2011). The expression of CCR5 or its ligands is increased in AD, and inactivation or deficiency of CCR5 reduces inflammation and enhances cognition (Necula et al., 2021). TSPO is a major indicator of neuroinflammation and has been implicated in the pathogenesis and progression of many neurodegenerative diseases, including AD. TSPO expression in the brain is low under physiological conditions, but it is upregulated in response to glial activation. TSPO, which is an *in vivo* marker of neuroinflammation, is significantly upregulated in AD and has now been used in neuroimaging (Tournier et al., 2020). These phytochemicals of AO that target molecules that are related to neuroinflammation deserve more attention in follow-up studies.

## Conclusion

In conclusion, our study provides a powerful integrated network medicine strategy to understand the biological basis of kidney-essence deficiency in neurodegenerative dementia and the targets of AO based on several important pathophysiological processes related to neurodegenerative dementia, including ACh metabolism, Aβ aggregation, tau accumulation, neuroinflammation, and neuron loss (Figure 10). Twenty-six bioactive phytochemicals of AO were found to target 168 key molecules in neurodegenerative dementia. Yakuchinone B, 5-HYD, oxyhylladiketone, oxyphyllacinol, butyl-β-D-fructopyranoside, dibutyl phthalate, chrysin, yakuchinone A, rhamnetin, and rhamnocitrin were identified as the key phytochemicals of AO that regulate the pathogenesis of neurodegenerative dementia in a multitargeted manner. In addition, this approach of studying the pharmacological mechanism of medicinal plants and the biological basis of TCM syndrome may be helpful in studying the translation of TCM.

## Data availability statement

The original contributions presented in this study are included in the article/Supplementary material, further inquiries can be directed to the corresponding authors.

## Author contributions

PZ, JM, and QT: conceptualization. PZ: investigation and writing—original draft. Y-CL, H-FS, S-WQ, Y-WS, and C-YY: methodology. QT and ML: supervision. PZ, X-MW, YW, and Y-NL: visualization. QT and Y-CW: writing—review and editing. All authors read and agreed to the published version of the manuscript.

## Footnotes

1
http://www.ncbi.nlm.nih.gov/pubmed

2
https://pubchem.ncbi.nlm.nih.gov/

3
http://www.icdrug.com/ICDrug/ADMET

4
http://www.swissadme.ch/

5
https://tox-new.charite.de/protox_II/

6
http://www.swisstargetprediction.ch/

7
http://www.ncbi.nlm.nih.gov/geo/

8
http://www.alzdata.org/

9
http://www.genome.jp/kegg/

10
http://www.bioinformatics.com.cn/

11
https://cn.string-db.org/

12
https://bioinfogp.cnb.csic.es/tools/venny/index.html

13
http://www.lephar.com/software.htm

14
https://www.rcsb.org/

15
https://demos.mn-am.com/corina.html

16
http://herb.ac.cn/

17
www.icdrug.com/ICDrug/A

## Supplementary material

Supplementary Figure 1 Cell types of 282 targets of AO for AD treatment located in the human brain. **(A)** Venn diagram showing the intersection of AO targets localized to neurons, astrocytes, and microglia. **(B)** 34, 23, and 8 AO targets were specifically located in brain neurons, microglia, and astrocytes, respectively.

Supplementary Figure 2 The PPI network was constructed for the 105 potential targets of AO associated with tau (encoded by MAPT) phosphorylation. Red circle node: MAPT. Nodes in cyan and green represent AO targets that directly or indirectly interact with MAPT, respectively.

Supplementary Figure 3 The 2D chemical structure of AO phytochemicals in Figure 9. Numbers in red fonts indicated the synthetic accessibility score (SAscore, less than 6 implies easy to synthesize), which is calculated using ADMETlab 2.0 (https://admetmesh.scbdd.com/).

Supplementary Figure 4 The Sankey diagram showed that 2, 18, 11, and 17 of the AO targets regulating Aβ production and degradation, tau phosphorylation, neuroinflammation, and neuron death were core anti-AD targets. The core targets refer to the top 30 targets ranked by the degree value of the PPI network of 282 AO anti-AD targets.

## Abbreviations

5-HYD: 5-hydroxy-1,7-bis (4-hydroxy-3-methoxyphenyl) heptan-3-one
AO: *Alpinia oxyphylla* Miquel
ACh: acetylcholine
ACHE: acetylcholinesterase
ALOX5: 5-lipoxygenase
AD: Alzheimer’s disease
Aβ: amyloid-β
BBB: blood brain barrier
BCHE: butyrylcholinesterase
BH: Benjamini–Hochberg
BP: biological process
CDK5: cyclin-dependent kinase 5
DEGs: differentially expressed genes
DO: disease ontology
GEO: gene expression omnibus
GO: gene ontology
GSK3β: glycogen synthase kinase 3
Hacc: hydrogen bond acceptors
Hdon: hydrogen bond donors
HOB: human oral bioavailability
LogP: lipid-water partition coefficient
MW: molecule weight
NFTs: neurofibrillary tangles
PCA: principal component analysis
PPI: protein–protein interaction
Q-marker: quality marker
Rbon: rotatable bonds
RO5: Lipinski’s rule of five
SPs: senile plaques
TCM: traditional Chinese medicine
UHPLC-MS/MS: ultra-high-performance liquid chromatography with triple quadrupole mass spectrometry.
