# Supplementary figures and images for: Targets and mechanisms of Alpinia oxyphylla Miquel fruits in treating neurodegenerative dementia
Source: Front Aging Neurosci. 2022 Nov 30;14:1013891. doi: 10.3389/fnagi.2022.1013891 (PMC9749063; doi:10.3389/fnagi.2022.1013891)

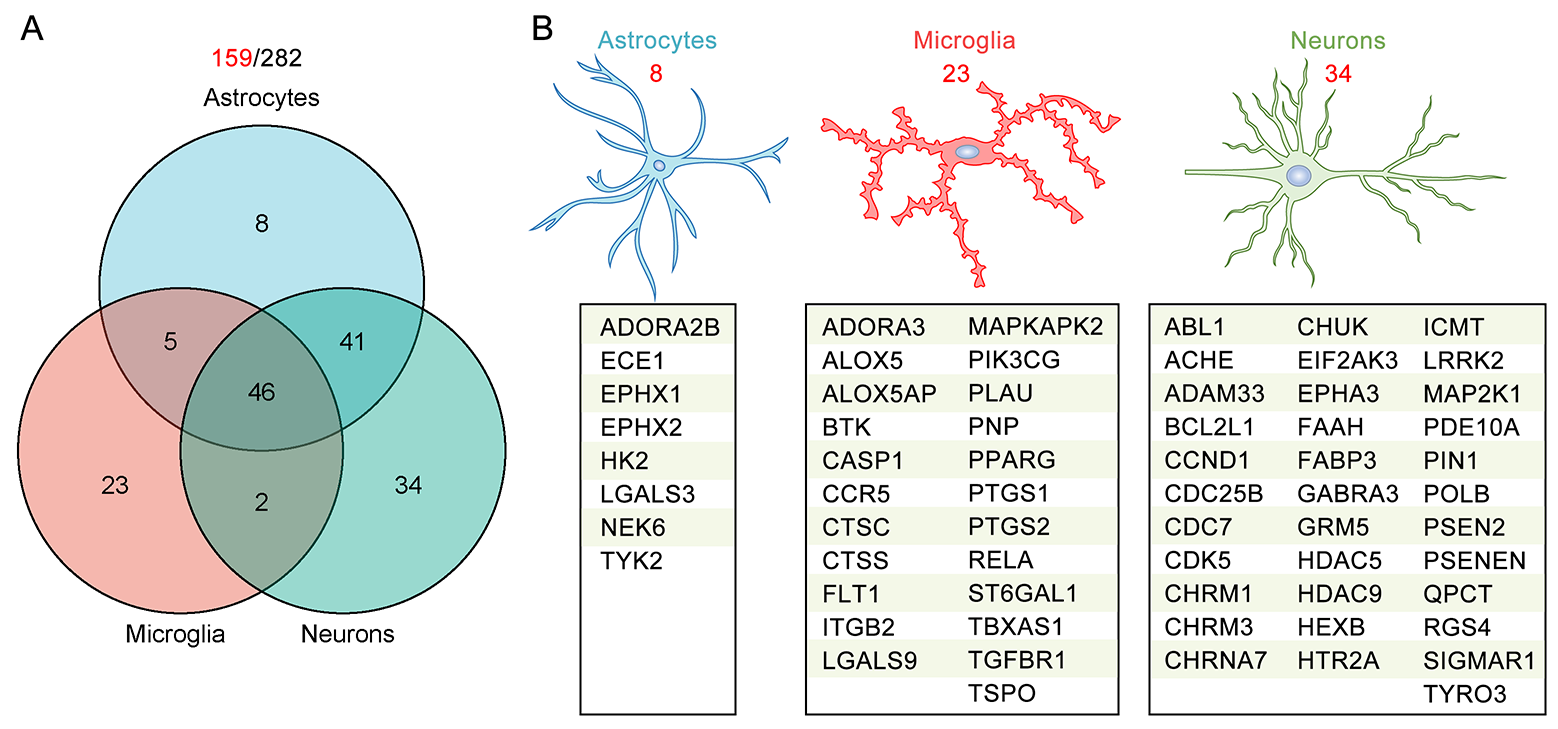

Supplement: Supplementary Figure 1 — Cell types of 282 targets of AO for AD treatment located in the human brain. (A) Venn diagram showing the intersection of AO targets localized to neurons, astrocytes, and microglia. (B) 34, 23, and 8 AO targets were specifically located in brain neurons, microglia, and astrocytes, respectively. [file Image_1.TIF]

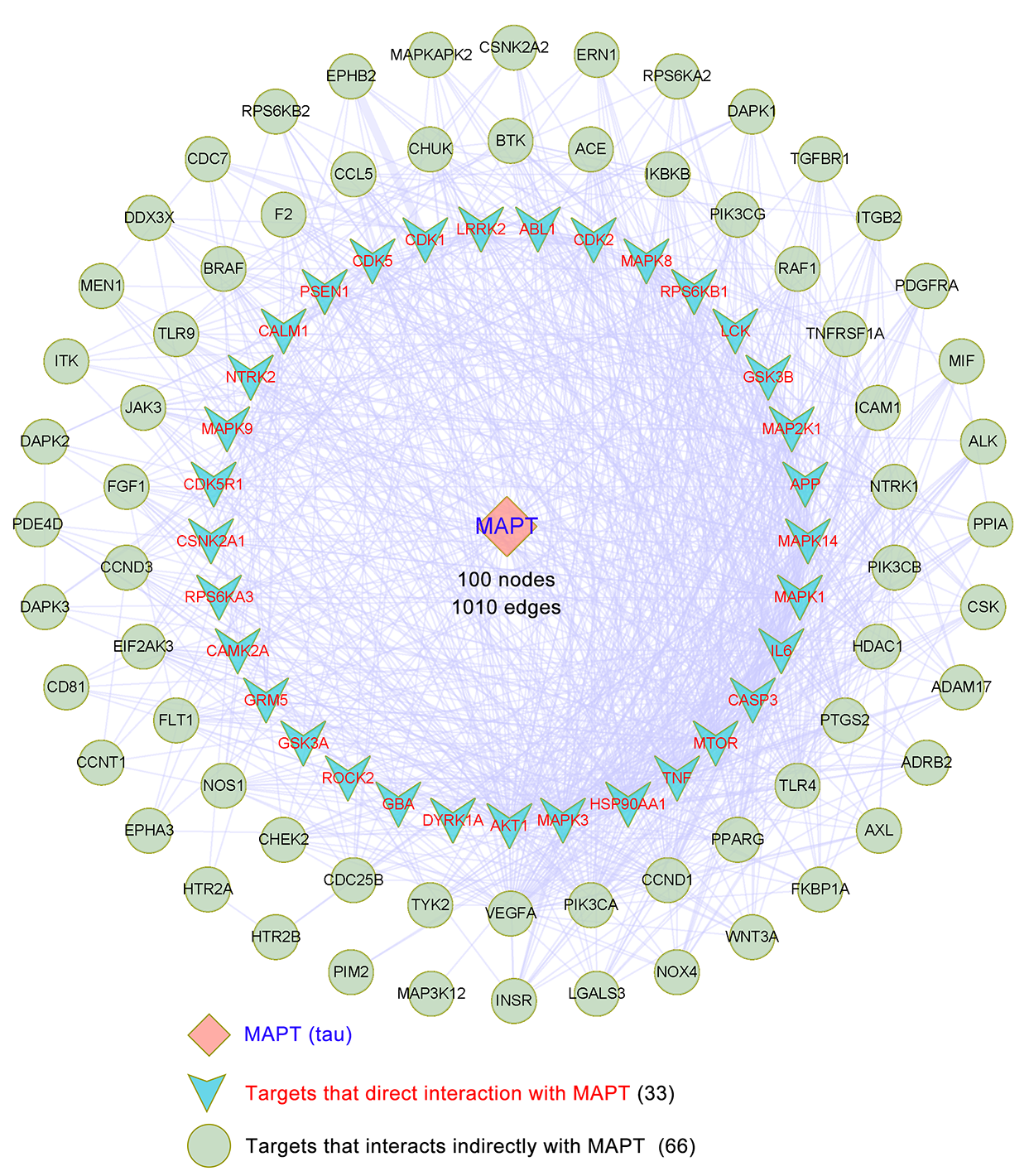

Supplement: Supplementary Figure 2 — The PPI network was constructed for the 105 potential targets of AO associated with tau (encoded by MAPT) phosphorylation. Red circle node: MAPT. Nodes in cyan and green represent AO targets that directly or indirectly interact with MAPT, respectively. [file Image_2.tif]

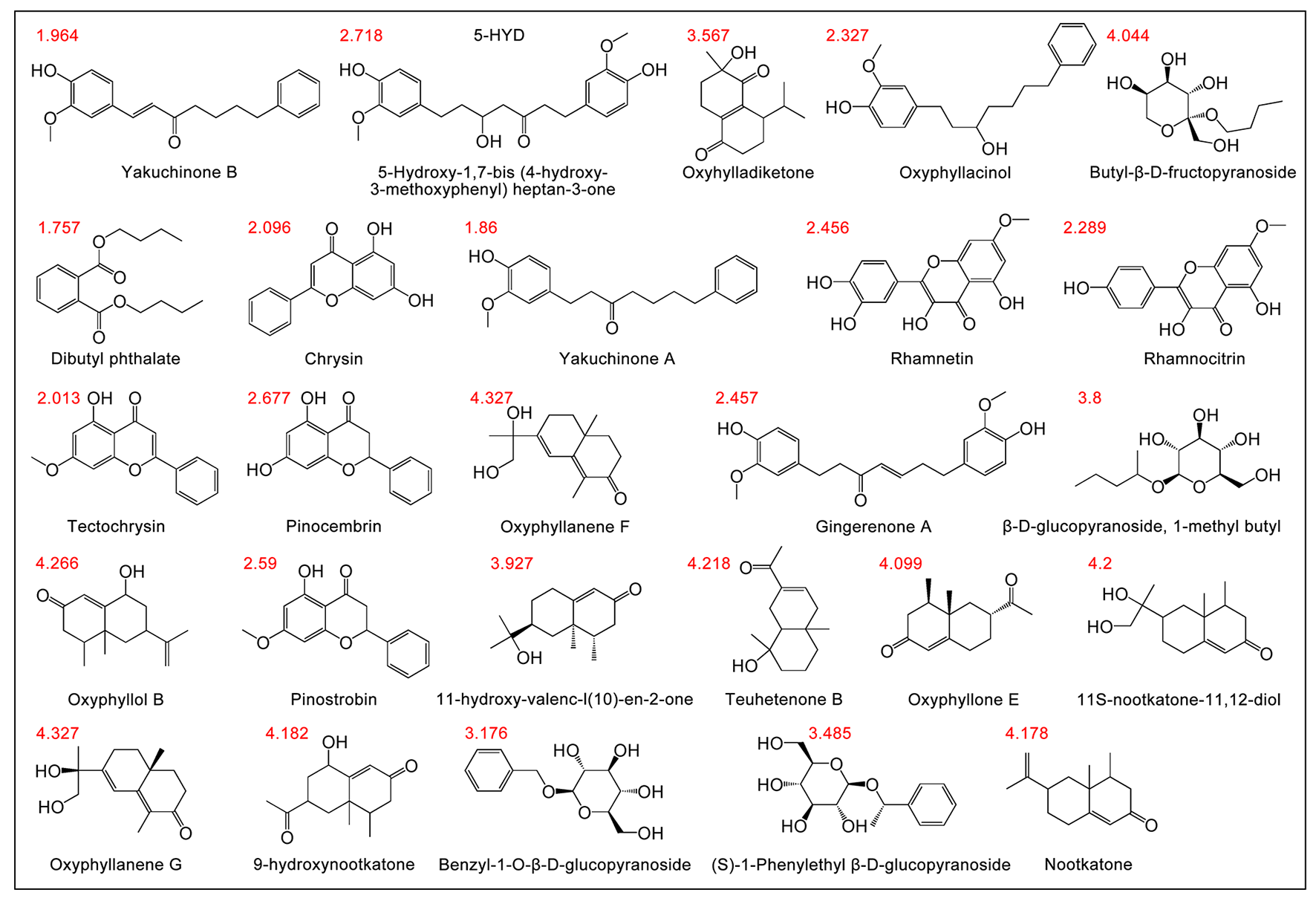

Supplement: Supplementary Figure 3 — The 2D chemical structure of AO phytochemicals in Figure 9. Numbers in red fonts indicated the synthetic accessibility score (SAscore, less than 6 implies easy to synthesize), which is calculated using ADMETlab 2.0 (https://admetmesh.scbdd.com/). [file Image_3.TIF]

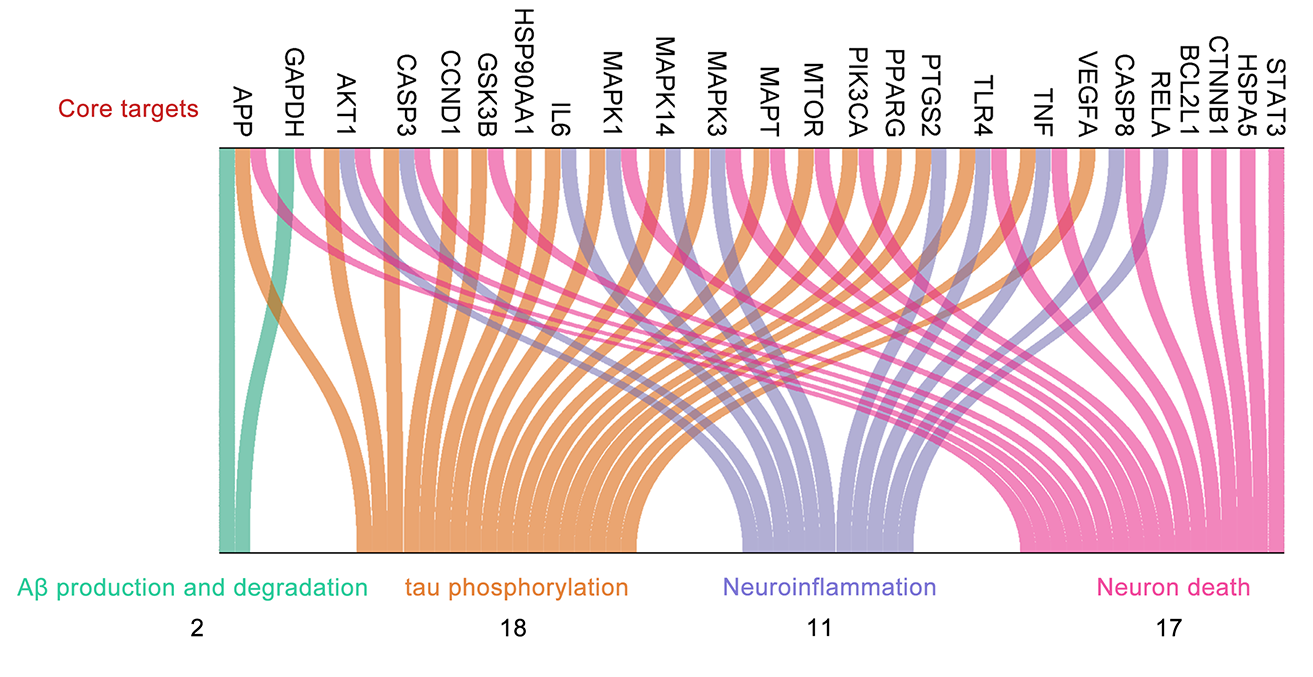

Supplement: Supplementary Figure 4 — The Sankey diagram showed that 2, 18, 11, and 17 of the AO targets regulating Aβ production and degradation, tau phosphorylation, neuroinflammation, and neuron death were core anti-AD targets. The core targets refer to the top 30 targets ranked by the degree value of the PPI network of 282 AO anti-AD targets. [file Image_4.TIF]
